# Return to Learn: Academic Effects of Concussion in High School and College Student-Athletes

**DOI:** 10.3389/fped.2020.00057

**Published:** 2020-03-04

**Authors:** Acacia Holmes, Zhongxue Chen, Lilian Yahng, David Fletcher, Keisuke Kawata

**Affiliations:** ^1^Department of Kinesiology, School of Public Health-Bloomington, Indiana University, Bloomington, IN, United States; ^2^Department of Epidemiology and Biostatistics, School of Public Health-Bloomington, Indiana University, Bloomington, IN, United States; ^3^Center for Survey Research, Indiana University, Bloomington, IN, United States; ^4^Indiana University Health Center, Indiana University, Bloomington, IN, United States; ^5^Program in Neuroscience, College of Arts and Sciences, Indiana University, Bloomington, IN, United States

**Keywords:** mild traumatic brain injury, sport-related concussion, sports injury, brain injury, high school

## Abstract

While awareness and understanding of concussion have improved drastically, post-concussion management in academic settings is still at its infancy. The aim of the study was to examine to what extent concussion influences academic performance and to whether there would be a difference in concussion effects on academic performance between high school and college students. This cross-sectional survey study included students, who were between 14 and 24 years old and sustained a sport-related concussion within the previous year. The study used a modified chain-referral sampling method, by distributing a questionnaire link to 3,000 randomly stratified athletic trainers, who worked in high school or college settings. These athletic trainers were then asked to forward the questionnaire link to students in their team, who have sustained a concussion within the previous year. The questionnaire recorded responses regarding demographics (age, sex, race/ethnicity, number of previous concussions); 22 concussion-related symptoms in a binary scale (presence/absence); perception of difficulties in math, reading, writing, computer use, and attention in a 5-point Likert scale; and asymptomatic duration of academic engagement in a 7-point Likert scale. There were 130 respondents with a history of concussion in the past year (*n* = 59 high school, *n* = 71 college). While recovering from concussion, significantly more college students (84.5%) reported “difficulty concentrating” than high school students (68.6%: *p* = 0.049). High -school students experienced more difficulty with math than college students (*p* = 0.002), whereas college students experienced more difficulty with reading (*p* = 0.013) and computer use (*p* = 0.026) than high school students. Asymptomatic duration of cognitive activity was influenced by age (*p* = 0.0004), where younger students were less tolerant in performing academic tasks after a concussion than older students. Our data indicate that concussions can induce negative symptoms in the academic setting regardless of age. The post-concussion difficulties in academic performance may be a grade-dependent manner, where concussions triggered difficulty in math among high school students and in reading and computer use among college students. It is clear that there is a need for guidelines and accommodations to support students with concussion in academic settings, and the guideline should reflect the age-dependent response to concussions.

## Introduction

A concussion is classified as a mild form of traumatic brain injury (mTBI), but changes in neural function following concussions are far from benign. The residual concussion effects can impede sports participations, social relationships, and academic activities (1–3). Concussions are often regarded as an invisible disability, given that concussion symptoms such as headache, dizziness, blurred vision, and difficulty concentrating can be concealable and remain unnoticed (4, 5). Unlike athletic settings where coaches and sports medicine staffs witness concussion events and implement individualized treatments (6, 7), athletes with a concussion may largely be expected to “keep up” with their academic responsibilities. However, post-concussion management in academic settings is often neglected, and the current “return-to-learn” (RTL) guideline is based on experts' suggestions at best (8–10).

The concept of RTL was first introduced in 2010 by Dr. Neal McGrath (11), who modeled the rapidly developing return-to-play protocol (1, 6, 12). The preliminary RTL protocol was supported by the prospective data from Ransom et al. (13), who demonstrated that a concussion can negatively impact one's perception toward academic tasks (e.g., difficulty in taking notes, studying, and understanding class materials). The difficulties in perception toward linguistic and math/science classes were age-dependent, with the majority of high school students expressing their concern in these classes compared to middle school and elementary school students during their recovery from a concussion. These data played a catalytic role in a recent pilot longitudinal study that revealed better sleep and more water consumption to be ameliorating factors for concussion recovery in academic settings, whereas longer music time and lack of physical activity delayed symptom resolution (14). However, the difference in post-concussion academic well-being between high school and college students has never been presented.

Therefore, the present cross-sectional survey study aimed to examine the prevalence of concussion-related symptoms in classroom settings. The study assessed perceptions on fundamental academic skills (computer, math, reading, writing, and attention) in relation to key demographic factors (level of schooling and age) while recovering from a concussion. Lastly, the duration in which one can engage in cognitive activity without experiencing concussion symptoms was examined. There were three hypotheses in this study: (1) While concussion symptoms would be prevalent in academic settings, there would be no differences in concussion symptom levels between high school and college students; (2) There would be significant differences in perceptions on fundamental academic skills between high school and college students; and (3) There would be a significant positive relationship between age and the duration of asymptomatic cognitive activity.

## Materials and Methods

### Patients

This cross-sectional online survey study used a modified chain-referral sampling method to obtain responses from concussion patients. The sample consisted of survey responses from 130 students, who have experienced a concussion from sports activity. Inclusion criteria were (1) students being between 14 and 24 years old, who were enrolled in school and participated in organized sports at the time of their concussion, (2) participants must have sustained a sport-related concussion within 1 year of the date that he/she took the survey, and (3) participants who sustained a sport-related concussion must have been diagnosed by a healthcare professional (e.g., physician or athletic trainer). Participants who did not participate in organized sports and/or have never been diagnosed with a sport-related concussion were excluded from the study. The study was approved by the Indiana University Institutional Review Board.

### Study Procedure

In order to control subjects' recall bias, a cutoff duration of <1 year from concussion incidents was predetermined. Oftentimes, a concussion diagnosis in high school and college athletes is made by team/school physicians and athletic trainers, instead of seeking medical triage at a hospital. Therefore, athletic trainers were thought to be an ideal referrer since they are in close proximity to athletes' overall health management. Using the National Athletic Trainers' Association Survey Service Program, an e-mail containing a survey link was distributed to 3,000 randomly stratified certified athletic trainers, who were employed in a high school or college/university. In the e-mail, athletic trainers were asked to forward the e-mail with the survey link to students in their team who have sustained a concussion within the past year. The e-mail explicitly indicated that the survey does not inquire any identifiable information (e.g., name, address, e-mail address) to ensure complete anonymity.

### Instrumentation

An online questionnaire was developed by the authors and concussion experts using Qualtrics (Provo, UT). The questionnaire was adapted from previous studies on concussion management inside the classroom (13) and reflected published recommendations for cognitive rest (8, 11, 15). The questionnaire consisted of blocks including consent and demographics, sport participation, previous concussion history, concussion symptomatology in classroom settings, perception of difficulty/easiness toward academic tasks, and a duration of asymptomatic cognitive activity. The demographics block collected information regarding age, sex, ethnicity, level, and type of sports participation, and current academic enrollment. All the other blocks included binary choice (yes or no), 5- and 7-point Likert scale, and multiple choice.

Prior to dissemination, the questionnaire was distributed to content experts, including four sports medicine physicians, five clinical athletic trainers, five athletic training faculty members, three psychologists, two neurologists, and one survey methodologist. Each person was asked to validate the length of time needed to complete the survey (< 10 min). Survey items were rated on a scale of 1–5 for clarity and relevance (1-poor, 2-understandable, 3-good, 4-very good, 5-excellent). The questionnaire was revised based on the feedback until a consensus was met that the surveys contained clear and relevant questions. Following this step, a cohort of 10 high school and 10 college student-athletes with a history of concussion in the past year was recruited for a pilot study. The test–retest reliability of a 1-week interval showed a statistically significant intraclass correlation coefficient (ICC) in all questions (ICC range, 0.780–0.984; *p* < 0.017). Please see Supplementary File for an example of the questionnaire.

### Statistical Analysis

Group differences (high school vs. college) in demographics and concussion symptoms were tested using the independent *t*-test for age and history of concussion and chi-square (χ^2^) statistics for sex, race/ethnicity, and concussion symptoms. Due to statistically significant results from Shapiro–Wilk tests, we conducted a Mann–Whitney U test to evaluate 5-point Likert scale data concerning the perception of difficulty with academic tasks (math, reading, writing, computer use/projector screen, and attention) between high school and college. A follow-up Spearman's rank correlation coefficient (RCC) was used to explore the relationship between age and the perception of difficulty with the academic tasks. A Kruskal–Wallis H test was used to assess an age effect on asymptomatic duration of cognitive activity, followed by Spearman's RCC to assess the association between age and asymptomatic duration of cognitive activity. All the data were analyzed with SPSS version 25, and significance was set at *p* < 0.05.

## Results

### Demographics and Concussion Symptoms in Academic Settings

There was no statistically significant difference in demographic characteristics, except for ages, between high school and college students with concussions (Table 1). More college students (84.5%) reported difficulty in concentrating in academic settings than high school students (68.6%: *p* = 0.049; Table 1) after a concussion. It is also worth noting that ~90, 76, 57, and 54% of overall students with concussions reported the presence of headache, difficulty concentrating, sensitivity to light, and feeling slowed down in academic settings, respectively (Table 1).

**Table 1:** Demographic and concussion symptoms experienced in academic settings.

**Characteristic**	**High school**	**College**
*n*	59	71
Age, years, median (IQR)	16 (15–17)	20 (19–21)^**^
Male, *n* (%)	34 (57.6)	31^a^ (43.7)
Female, *n* (%)	25 (42.4)	39^a^ (54.9)
Race/ethnicity, *n* (%)		
White	49 (83)	50 (70.4)
Black/African-American	2 (3.4)	11 (15.5)
Hispanic	2 (3.4)	0 (0)
Asian	3 (5.1)	3 (4.2)
Multiracial	3 (5.1)	4 (5.6)
Other	0 (0)	3 (4.2)
History of Concussion, mean (SD)	1.92 (1.17)	1.72 (0.91)
Concussion Symptoms in Academic Setting, *n* (%)		
Headache	47 (92.2)	51 (87.9)
Dizziness	14 (27.5)	11 (19.0)
Feeling Slowed Down	29 (56.9)	31 (53.5)
Blurred Vision	7 (13.7)	8 (13.8)
Drowsiness	22 (43.1)	19 (32.8)
Nausea	6 (11.8)	9 (15.5)
Difficulty Concentrating	35 (68.6)	49 (84.5)^*^
Difficulty Remembering	21 (41.2)	21 (36.2)
Sensitivity to Light	28 (54.9)	36 (62.1)
Sensitivity to Noise	20 (39.2)	23 (39.7)

*IQR, interquartile range; SD, standard deviation*.

a One participant identified as college student but preferred not to answer for sex;

* p < 0.05,

** *p < 0.01*.

### Concussion Impacts on Individual Perception Toward Academic Tasks

Mann–Whitney *U*-tests were conducted to evaluate a perception of difficulty/easiness toward fundamental academic skills (math, reading, writing, engaging in computer/projector screen, and paying attention to teachers) between high school and college students with concussions. Students responded to the question, “How easy or difficult was it for you to do Math [for example] in class after your concussion?” High school students perceived more difficulty with math than college students (*p* = 0.002). On the other hand, college students perceived more difficulty with reading (*p* = 0.013) and engaging in computer/projector screen (*p* = 0.026) than high school students (Table 2). These results were supported by Spearman's RCC assessing the correlations between age and perceptions toward academic tasks. There was a significant positive correlation between age and math, indicating that performing math became easier with age (*r*_s_ = 0.200, *p* = 0.037). Conversely, there were significant negative correlations between age and reading (*r*_s_ = −0.259, *p* = 0.007) as well as engaging in computer/projector screen (*r*_s_ = −0.205, *p* = 0.033; Table 2).

**Table 2:** Perception of difficulty in performing academic tasks after a concussion as a function of level of schooling and age.

**Academic activities^a^**	**High school**	**College**	***U***	***p***	**Age**	
					***r_***s***_***	***p***
Math	2.00 (0.85)	2.53 (0.89)	913.5	0.002	0.200	0.037
Reading	2.25 (0.89)	1.89 (0.87)	1,007.5	0.013	−0.259	0.007
Writing	2.84 (0.88)	2.72 (0.89)	1,261.5	0.530	−0.097	0.314
Engaging in computer/projector screen	2.06 (0.99)	1.68 (0.85)	1,033.0	0.026	−0.205	0.033
Pay attention to teachers	2.18 (0.91)	1.94 (0.80)	1,163.0	0.167	−0.121	0.210

*Values are expressed as mean (SD)*.

a *mean values of 5-point Likert scale: 1, extremely difficult; 2, somewhat difficult; 3, neither easy nor difficult; 4, somewhat easy; 5, extremely easy*.

*U, results from Mann–Whitney U-tests*.

*r_s_, results from Spearman's rank correlation coefficient*.

### Duration of Cognitive Engagement Without Experiencing Concussion Symptoms

**Figure 1:**
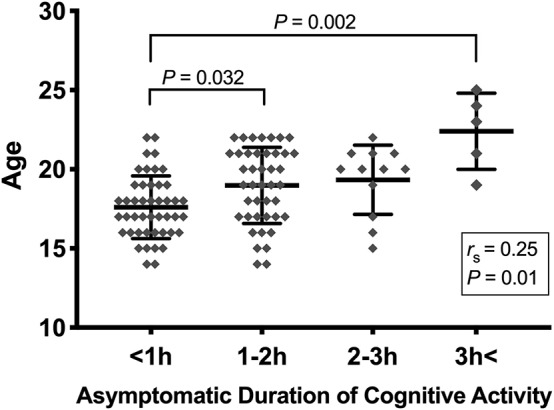
Asymptomatic duration of cognitive activity by age. The relationships between age and length of cognitive activity without experiencing concussion symptoms were evaluated by Kruskal–Wallis test. Significance difference was observed between less than 1 h and 1–2 h (*p* = 0.032), and between less than 1 h and more then 3 h (*p* = 0.002). The asymptomatic length of cognitive activity was positively correlated with age (*r*_s_ = 0.25, *p* = 0.01).

Students responded to the question, “How long were you able to do classwork-related activities each day before your concussion symptoms came back or became worse?” The Kruskal–Wallis H test showed that there was a statistically significant age effect on asymptomatic duration of cognitive activity (*H* = 18.35, *p* = 0.0004; Figure 1). Follow-up pairwise *post-hoc* comparisons with Bonferroni corrections revealed that the significant differences occurred between <1 h and 1–2 h (*p* = 0.032) and between <1 and >3 h (*p* = 0.002). This was supported by a significant positive correlation between age and asymptomatic duration of cognitive activity (*r*_s_ = 0.25, *p* = 0.01; Figure 1).

## Discussion

The present study investigated the concussion effects on students' well-being in academic settings in the context of the prevalence of concussion-related symptoms, perceptions of difficulty toward academic tasks, and the duration of academic engagement without experiencing symptoms. The data confirmed some previous findings and generated critical knowledge about concussion effects on classroom-related patients' well-being. First, concussion symptoms were vastly prevalent in academic settings regardless of age, with headache, difficulty concentrating, sensitivity to light, and feeling slowed down being their chief complaint. Second, there were differences in individual perceptions toward performing math, reading, and computer use between high school and college students. Lastly, older students were able to engage in academic tasks for longer periods of time without experiencing concussion symptoms, whereas younger students were unable to tolerate long duration of academic tasks without symptoms. To our knowledge, this is the first report indicating that age may have a significant influence on academic well-being after a concussion.

One of the critical findings from this study was that although the perception of difficulty in both reading and computer use increases with age, younger brains have less resiliency in conducting cognitive function for a longer period of time (Figure 1). This notion is in line with existing literature, whereby younger brains may become more sensitive to neurocognitive load while recovering from brain trauma. For instance, neurocognitive function in a young cohort (11–17 years) who had sustained a concussion exhibited substantial deficit in executive functioning and reaction time, which required up to 21 days to recover (16, 17). Abnormalities in emotional and somatic well-being have been observed among children 10–17 years of age, which persisted up to 5 weeks after brain trauma (18). Importantly, subclinical changes may linger in younger populations in response to one's interactions with society and the environment (19). A young brain undergoing injury responds to stimuli differently from a fully developed brain in such a way that young athletes with a concussion exhibit increased impulsive response as well as impairment in psychomotor speed and visual spatial skills (20–22).

The present study provides an important information on high school and college students' well-being after a concussion in academic settings. Consistent to the current consensus that the prevalence of concussion symptoms in academic settings is evident (23, 24), such that chief complaints including headache, difficulty concentrating, sensitivity to light, and feeling slowed down are critical inhibitors to engaging in neurocognitive function (25). These inhibitors might have played a significant role in attenuating one's ability to engage in fundamental academic tasks, including math, reading, and computer use. These data are consistent to the previous RTL study, which tested whether concussion symptoms (i.e., difficulty paying attention, cognitive fatigue, headache) led to impaired academic skills (i.e., increased time spent on homework, difficulty studying and understanding material) among elementary, middle, and high school students (13). Concussion effects were also apparent in academic subjects including math, language arts, and science, with which students' perceptions of difficulty were associated with the level of school. For example, high school students reported more difficulty in math, language arts, and science compared to middle and elementary school students. Middle school students had a more difficult time engaging in math, language arts, and science than elementary school students. One potential reason for our college student data with increased difficulty in reading and computer use compared to high school students may be due to the greater amount of reading, larger class settings with projector-based lectures, and more computer-based assignments. College students often have obligatory functions such as study halls, team meetings, and student organization, which collectively influence one's academic standing that ultimately affects academic scholarship, athletic eligibilities, and/or postgraduation paths. These obligatory functions impede adequate rest and quick recovery, leading to fatigue in central and peripheral neural processing regions (26, 27). As a result, college students tend to experience difficulty in concentration and comprehension of reading materials and increased sensitivity to light from a projector or computer screen.

There are several limitations to be noted in this study. Subjective reports, including concussion symptoms, difficulty in perception with academic activities, were not verified with school or medical records. Symptom reports could be subjected to one's delayed recall skill; however, our inclusion criterion of concussion incidence being <1 year reduces any potential variability in symptom recall. This study only examined students and lacked further information from school personnel, including teachers, nurses, and administrators. Assessing knowledge and awareness of concussions in these key personnel would further identify the need for academic accommodations and development of a RTL protocol. The current study, in conjunction with others, has delineated the prevalence of concussion effects on academic tasks, serving as a critical initial step to establish a stepwise RTL protocol.

## Conclusion

The results from this study indicate that concussions can induce concussion-related symptoms in the academic setting regardless of age. These symptoms, such as headache, difficulty concentrating, sensitivity to light, and feeling slowed down, can debilitate students' academic well-being, resulting in frustration and subsequent academic setbacks. It is clear that there is a need for guidelines and accommodations to support students with a concussion in academic settings, and the guideline should reflect the age-dependent response to concussions. Additionally, teachers' knowledge about concussions should be explored in order to expose the swelling knowledge gap. Researchers and clinicians are able to comprehensively establish a “return-to-learn” guideline based on empirical evidence.

## Data Availability Statement

The datasets generated for this study are available on request to the corresponding author.

## Ethics Statement

The studies involving human participants were reviewed and approved by Indiana University Institutional Review Board. Written informed consent from the participants' legal guardian/next of kin was not required to participate in this study in accordance with the national legislation and the institutional requirements.

## Author Contributions

AH and KK conceptualized and designed the study and the data collection instruments, collected data, drafted the initial manuscript, and reviewed and revised the manuscript. LY and ZC reviewed and revised the data collection instruments, carried out the initial analyses, and reviewed and revised the manuscript. DF designed the study, assisted in the clinical interpretation, and critically reviewed and revised the manuscript for important intellectual content. All authors have read and approved the final manuscript as submitted and agree to be accountable for all aspects of the work.

### Conflict of Interest

The authors declare that the research was conducted in the absence of any commercial or financial relationships that could be construed as a potential conflict of interest.
