# Supplementary material for: Return to Learn: Academic Effects of Concussion in High School and College Student-Athletes
Source: Front Pediatr. 2020 Mar 4;8:57. doi: 10.3389/fped.2020.00057 (PMC7065268; doi:10.3389/fped.2020.00057)
Supplement: Supplementary file 1 [file Data_Sheet_1.PDF]

Consent

Title of research study:
Return-to-Learn and Classroom Management of Sport-Related Concussion

Investigator and Department:
Acacia Holmes, LAT, ATC and Keisuke Kawata, PhD, ATC, Department of Kinesiology, Indiana University; and Lilian Yahng, MS, Center for Survey Research, Indiana University

Why are we doing this research?
Sport-related concussion is a mild form of traumatic brain injury that may distress cognitive function. The purpose of this study is to analyze the cognitive effects of concussion which may include: alterations in attention, memory and concentration. Furthermore, the study intends to uncover underlying perception in regards to high school and collegiate student-athletes' struggle in school and with classwork following sport-related concussion.

Why are you being invited to take part in this research study?
We have invited you to take part in this survey study because you are a student-athlete in high school or college who has been diagnosed with a sport-related concussion.

Who can I talk to?
If you have questions, concerns, or complaints, contact Acacia Holmes, LAT, ATC, School of Public Health, Indiana University, aaholmes@indiana.edu or Keisuke Kawata, PhD, ATC, School of Public Health, Indiana University, kkawata@indiana.edu, (812) 855-5244.

This research has been reviewed and approved by an Institutional Review Board (Protocol #1612412577). You may talk to them at 812-856-4242 or e-mail them at: IRB@IU.edu for any of the following:

- Your questions, concerns, or complaints are not being answered by the research team.
- You cannot reach the research team.
- You want to talk to someone besides the research team.
- You have questions about your rights as a research subject.
- You want to get information or provide input about this research.

How long does this survey last?
It takes approximately 5 minutes.

What are my responsibilities if I take part in this research?
There will be no responsibility regarding participation, but if you wish to participate in this study, we expect you to answer all questions honestly.

What happens if I say no, I do not want to be in this research?
You may decide not to take part in the research and it will not be held against you.

What happens if I say yes, but I change my mind later?
If you agree to take the survey and if you stop at any time, it will not be held against you.

What happens to the information we collect?
There will be no identifiable information collected in the survey. All the collected data will be kept in secured server and data will be only reported in terms of group differences.

If you agree to participate in the survey, please click yes and the arrow to begin.

Yes

No

Demographics

What year were you born?

Year

Gender

Male

Female

Other (please specify):

Prefer not to answer

What is your ethnicity?

White

Black or African American

American Indian or Alaska Native

Asian

Native Hawaiian or Pacific Islander

Hispanic

Multiracial

Other

Prefer not to answer

Are you a high school or college student?

High School

College

Do you participate in organized sports?

Yes

No

What level of sport do you currently participate in? Select all that apply.

High school

Club

What level of sport do you currently participate in? Select all that apply.

Intramural

Club

Intercollegiate

What sports do you participate in? Select all that apply.

Baseball

Basketball

Cheer

Football

Golf

Gymnastics

Ice Hockey

Lacrosse

Rugby

Soccer

Softball

Swimming/Diving

Tennis

Track and Field/Cross Country

Volleyball

Wrestling

Other:

Do you have a certified Athletic Trainer at your school or organization?

Yes

No

Have you ever been diagnosed with a concussion?

Yes

No

Not sure

Who diagnosed you with a concussion?

Physician

Certified Athletic Trainer

School nurse

Parent(s)

Coach

Other

How many sport-related concussions have you had?

1

2

3

4

5

6+

How long ago was your most recent concussion?

Month

Year

Please identify your symptoms during the time of your most recent concussion. Select all that apply.

Difficulty concentrating

Difficulty remembering

Sensitivity to noise

Headache

Feeling slowed down

Dizziness

Nausea

Blurred vision

Other (please specify):

Sensitivity to light

Drowsiness

Loss of consciousness

Did you experience concussion symptoms in school and/or while doing classwork?

Yes

No

Which of the following concussion symptoms were most noticeable in school and/or while doing classwork? Select all that apply.

Sensitivity to noise

Difficulty remembering

Nausea

Drowsiness

Blurred vision

Other (please specify):

Difficulty concentrating

Dizziness

Sensitivity to light

Feeling slowed down

Headache

Which classwork-related activities made your concussion symptoms worse? Select all that apply.

Math

Reading

Writing

Computer use

Other (please specify):

None

How difficult or easy was it to do MATH while recovering from your concussion?

Extremely difficult

Somewhat difficult

Neither easy or difficult

Somewhat easy

Extremely easy

How difficult or easy was it to READ while recovering from your concussion?

Extremely difficult

Somewhat difficult

Neither easy or difficult

Somewhat easy

Extremely easy

How difficult or easy was it to WRITE while recovering from your concussion?

Extremely difficult

Somewhat difficult

Neither easy or difficult

Somewhat easy

Extremely easy

How difficult or easy was it to USE A COMPUTER or LOOK AT A PROJECTOR SCREEN while recovering from your concussion?

Extremely difficult

Somewhat difficult

Neither easy or difficult

Somewhat easy

Extremely easy

How difficult or easy was it to PAY ATTENTION to your teacher while recovering from your concussion?

Extremely difficult

Somewhat difficult

Neither easy nor difficult

Somewhat easy

Extremely easy

How long were you able to do classwork-related activities each day before your concussion symptoms came back or became worse?

Less than 1 hour

1-2 hours

2-3 hours

3-4 hours

4-5 hours

5-6 hours

More than 6 hours

Powered by Qualtrics
